# Microwave-Assisted One-Pot Synthesis of Pyrazolone Derivatives under Solvent-Free Conditions

**DOI:** 10.3390/molecules15053593

**Published:** 2010-05-17

**Authors:** Ruoqun Ma, Jin Zhu, Jie Liu, Lili Chen, Xu Shen, Hualiang Jiang, Jian Li

**Affiliations:** 1 School of Pharmacy, East China University of Science and Technology, 130 Mei Long Road, Shanghai 200237, China; 2 State Key Laboratory of Drug Research, Shanghai Institute of Materia Medica, Chinese Academy of Sciences, 555 Zuchongzhi Road, Shanghai 201203, China; 3 Drug Discovery and Design Center, Shanghai Institute of Materia Medica, Chinese Academy of Sciences, 555 Zuchongzhi Road, Shanghai 201203, China

**Keywords:** microwave-assisted reactions, one-pot, pyrazolones

## Abstract

An efficient one-pot method to generate structurally diverse and medicinally interesting pyrazolone derivatives in good to excellent yields of 51–98% under microwave irradiation and solvent-free conditions has been developed.

## 1. Introduction

**Figure 1:**
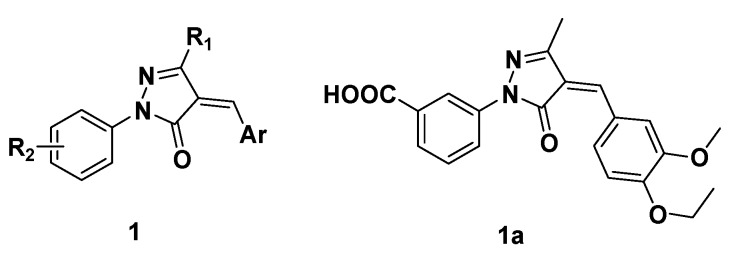
The structures of 4-arylidenepyrazolone derivatives **1** and FXR antagonist **1a**.

Pyrazolone derivatives are an important class of heterocyclic compounds that occur in many drugs and synthetic products [1]. These compounds exhibit remarkable antitubercular [2,3], antifungal [4,5], antibacterial [6], anti-inflammatory [7], and antitumor activities [8]. In our effort to identify new farnesoid X receptor (FXR) ligands [9], we recently found by virtual screening that a 4-arylidene-pyrazolone derivative **1a** (Figure 1) was a FXR antagonist (unpublished data). Consequently, to explore the structure activity relationships (SAR) for this family of compounds, a facile and practical approach for synthesizing 4-arylidenepyrazolone-containing derivatives **1** (Figure 1) became desirable.

**Scheme 1:**
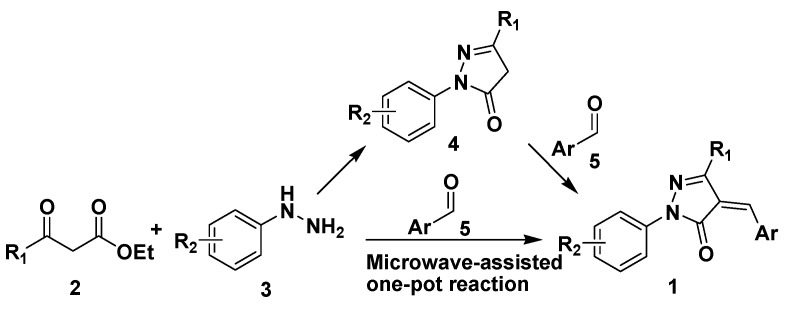
The synthetic routes to 4-arylidenepyrazolone derivatives **1** by classical two step or the proposed one-pot method.

Generally, 4-arylidenepyrazolone derivatives **1** are synthesized using as starting materials substituted aldehydes **5** [10,11,12] (or their their acetal [13,14] or imine [15,16] precursors) and 2-pyrazolin-5-ones **4**, the latter generally being obtained by the Knorr condensation [17,18] of β-ketoesters **2** with substituted hydrazines **3** (Scheme 1). It is obvious that all described methods involve multiple step reactions and most of them produce equimolar amounts of unwanted by-products. In this respect, the development of a one-pot reaction using readily available chemicals would be of considerable significance due to its synthetic efficiency and atom economy.

Microwave-assisted organic reactions have been applied to a wide range of reaction types [19,20,21,22,23,24,25], especially cycloaddition reactions. Microwaves accelerate a variety of synthetic transformations providing time- and energy-saving protocols [19,20,21,22,23,24,25]. Recently, Botta *et al.* [26]. reported a microwave-assisted three-component Knoevenagel/hetero Diels–Alder reaction, which suggested that 2-pyrazolin-5-ones **4** might react well with aldehydes **5** under microwave irradiation conditions. Inspired by this reaction, we envisioned that a new three-component (compounds **2**, **3**, and **5**) one-pot reaction might take place using a microwave-assisted approach and would directly create the target 4-arylidene-pyrazolone scaffolds **1** (Scheme 1).

## 2. Results and Discussion

### 2.1. Optimization of the reaction conditions

Initially we selected ethyl acetoacetate (**2a**), 3-nitrophenylhydrazine (**3b**) and 3-methoxy-4-ethoxy-benzaldehyde (**5a**) as the model substrates for the optimization of the reaction conditions, which included microwave oven power, time of irradiation, solid supports and reactant ratios. The results are summarized in Table 1-Table 2. The preliminary investigations revealed that the one-pot reaction occurred, as designed. Nevertheless, the reaction efficiency was highly microwave oven power dependent (entries 1-3, Table 1), with a good yield being achieved at 420 W. We then compared the synthesis of **1b** with irradiation times of 5 min, 10 min, and 15 min at 420 W, and the corresponding yields were 54%, 71%, and 62%, respectively (entries 2 and 4,5, Table 1). 

**Table 1:** Optimization of the power of microwave oven and time of irradiation.*^a^*

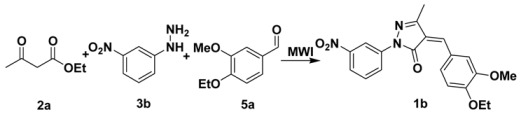			
Entry	Power (W)	Time (min)	Yield (%)*^b^*
1	280	5	20
2	420	5	67
3	560	5	54
4	420	10	71
5	420	15	62

*^a^* the reaction was carried out with **2a, 3b **and **5a **in equimolar ratio (0.3 mmol); for the detailed reaction conditions see the Experimental section; *^b ^*Isolated yields.

**Table 2:** Optimization of the reactant’s equivalence ratio and solid support.*^a^*

Entry	Solid support	Reagent Ratio			Yield (%)*^b^*
		2a	3b	5a	
1	-	1	1.2	1	60
2	-	1.2	1	1	78
3	-	1	1	1.2	63
4	-	1.5	1	1	83
5	-	2	1	1	73
6	-	2.5	1	1	79
7	-	3	1	1	81
8	SiO_2_	1.5	1	1	81
9	Al_2_O_3_	1.5	1	1	80

*^a^* The reaction was carried out under microwave radiation 420 W for 10 min; for detailed reaction conditions see the Experimental section; *^b ^*Isolated yields.

Encouraged by the promising results, we further optimized the reactant ratio (entries 1–7, Table 2) and solid support (entries 8,9, Table 2). The optimum results were obtained when the reactants were mixed is a ratio of **2a**/**3b**/**5a** = 1.5/1/1 without solid support and irradiated at 420 W for 10 min (entry 4, Table 2). Solid supports such as aluminium oxides and silica gel have been widely used in microwave-assisted organic synthesis to enhance substrates absorption of microwave energy. However, solid supports appeared to offer no advantages in our one-pot reaction (entries 4, and 8,9, Table 2).

### 2.2. Scope of microwave-assisted one-pot synthesis of 4-arylidenepyrazolone derivatives

Having established the optimal reaction conditions, we subjected a series of β-ketoesters, hydrazines, and aldehydes to them to explore the generality and scope of the one-pot process. As shown in Table 3, we were pleased to find that this method was applicable to a broad range substrate of substituted β-ketoester (**2a**-**b**), hydrazine (**3a**-**j**), and aldehyde (**5a**-**h**) substrates. 

**Table 3:** Scope of microwave-assisted one-pot synthesis of 4-arylidenepyrazolone derivatives.*^a^*

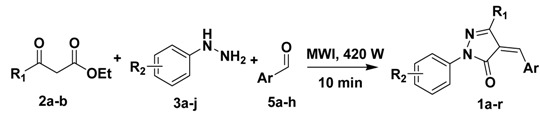				
Compd.	R_1_	R_2_	Ar	Yield (%)*^b^*
**1a**	Me, **2a**	3-CO_2_H, **3a**	3-MeO-4-EtO-Ph, **5a**	98
**1b**	Me, **2a**	3-NO_2_, **3b**	3-MeO-4-EtO-Ph, **5a**	83
**1c**	Me, **2a**	4-NO_2_, **3c**	3-MeO-4-EtO-Ph, **5a**	78
**1d**	Me, **2a**	4-CF_3_, **3d **	3-MeO-4-EtO-Ph, **5a**	53
**1e**	Me, **2a**	3-CF_3_, **3e**	3-MeO-4-EtO-Ph, **5a**	67*^c^*
**1f**	Me, **2a**	3,5-di-CF_3_, **3f**	3-MeO-4-EtO-Ph, **5a**	54*^c^*
**1g**	Me, **2a**	2-F, **3g**	3-MeO-4-EtO-Ph, **5a**	51
**1h**	Me, **2a**	3,4-di-Cl, **3h**	3-MeO-4-EtO-Ph, **5a**	86
**1i**	Me, **2a**	3,5-di-Cl, **3i**	3-MeO-4-EtO-Ph, **5a**	73*^c^*
**1j**	Me, **2a**	3-CO_2_H, **3a**	3-MeO-4-OH-Ph, **5b**	68
**1k**	Me, **2a**	3-CO_2_H, **3a**	3,4-di-OH-Ph, **5c**	73
**1l**	Me, **2a**	3-NO_2_, **3b**	5-Me-thiophen-2-yl, **5d**	53
**1m**	Me, **2a**	H, **3j**	3-MeO-4-EtO-Ph, **5a**	63
**1n**	Me, **2a**	H, **3j**	4-Br-Ph, **5e**	53*^c^*
**1o**	Ph, **2b**	3-NO_2_, **3b**	3-MeO-4-OH-Ph, **5c**	61
**1p**	Me, **2a**	3-CO_2_H, **3a**	3-MeO-4-PhCH_2_O-Ph, **5f**	84
**1q**	Me, **2a**	3-CO_2_H, **3a**	3-MeO-4-Me(CH_2_)_4_O-Ph, **5g**	83
**1r**	Me, **2a**	3-CO_2_H, **3a**	3-MeO-4-Me_2_CHO-Ph, **5h**	76

*^a ^*Unless stated otherwise, the reaction was carried out with **2** (0.45 mmol), **3** (0.3 mmol) and **5** (0.3 mmol) under microwave radiation 420 W for 10 min; for the detailed reaction conditions see the Experimental section; *^b ^*Isolated yields; *^c ^*These reactions were carried out with **2 **(0.36 mmol), **3** (0.3 mmol) and **5** (0.3 mmol).

The target products **1a**-**r** were prepared in good to excellent yields (51–98%). A variety of substituents on the aryl ring including halogens, -CO_2_H, -NO_2_, -CF_3_, alkoxy, *etc*. were well tolerated. These outcomes imply that electronic features have a marginal effect on the process. Examination of the results also revealed that the steric effects also play a minimal role in governing the reaction efficiency, as all *para*- (such as compound **1c**), *meta*- (such as compound **1e**), and *ortho*- (such as compound **1g**) substituted substrates were smoothly transformed into the desired products. Heterocycles (compound **1l**) could efficiently participate in the one-pot reaction as well. Finally, it is noteworthy that the process features simple operation and purification, and all target products could be directly obtained by simple suction filtration and washing with ethyl acetate.

**Figure 2:**
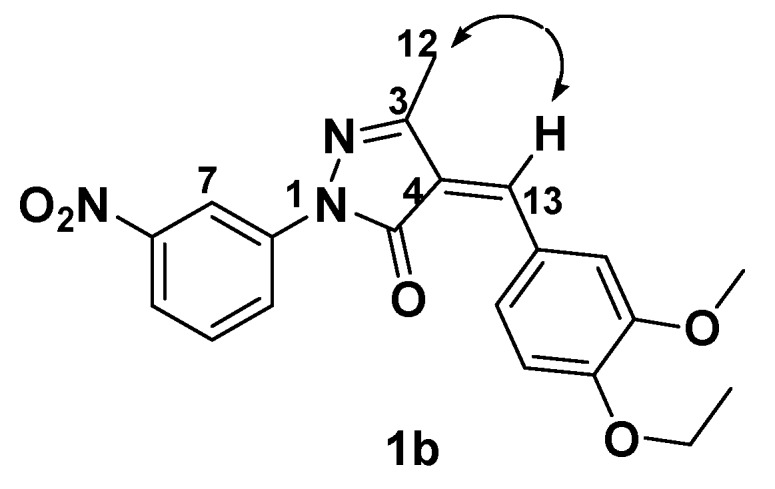
NOE correlation (double-headed arrows) established the stereochemistry of the double bond in **1b**.

With regard to the stereochemistry of the 4-arylidenepyrazolone products **1a**-**r**, they were all exclusively assigned the *Z*-configuration based on NOE spectroscopy of compound **1b**. NOE was observed between H-13 (*δ* 7.37) and H-12 (*δ* 2.38), suggesting a *Z* configuration at Δ^[4,13]^ (Figure 2).

## 3. Experimental

### 3.1. General

The reagents were purchased from Shanghai Chemical Reagent Company, Lancaster, and Acros, and used without further purification. Yields were not optimized. Nuclear magnetic resonance (NMR) spectra were recorded on a Brucker AMX-400 NMR instrument (^1^H at 400 MHz and ^13^C at 100 MHz, respectively). Chemical shifts are reported in parts per million (ppm, δ) downfield from tetramethylsilane (TMS) used as internal standard. Proton coupling patterns were described as singlet (s), doublet (d), triplet (t), quartet (q), multiplet (m), and broad (br). Low- and high-resolution mass spectra (LRMS and HRMS) were obtained by electron ionization (EI) on a Finnigan MAT-95 instrument. Microwave experiments were carried out in a domestic microwave oven (Midea MM721AAU-PW).

### 3.2. General Procedure for the Preparation of ***1a-r***: Preparation of ***1b***

*(Z)-4-(4-Ethoxy-3-methoxybenzylidene)-3-methyl-1-(3-nitrophenyl)-pyrazolone *(**1b**). A one-neck 50-mL flask containing ethyl acetoacetate (**2a**, 0.45 mmol), 3-nitrophenylhydrazine (**3b**, 0.3 mmol) and 3-methoxy-4-ethoxy-benzaldehyde (**5a**, 0.3 mmol) was placed in a domestic microwave oven and irradiated at a power of 420 W for 10 min. The solid obtained after cooling was triturated with ethyl acetate and collected by suction filtration, to afford product **1b** (95 mg, yield 83%) as an orange solid. m.p. 212–213 ºC. ^1^H-NMR (CDCl_3_): *δ* 1.55 (t, 3H), 2.38 (s, 3H), 4.07 (s, 3H), 4.27 (q, 2H), 6.98 (d, 1H), 7.37 (s, 1H), 7.59 (t, 1H), 7.75 (d, 1H), 8.03 (d, 1H), 8.47 (d, 1H), 8.93 (d, 2H); ^13^C-NMR (CDCl_3_): *δ* 162.7, 153.6, 152.1, 149.0, 148.6, 148.3, 139.6, 130.9, 129.6, 126.5, 124.0, 123.9, 118.8, 115.8, 113.5, 111.4, 64.7, 56.4, 14.6, 13.5; EI-MS m/z 381 (M^+^, 100%); HRMS (EI) m/z calcd. C_20_H_19_N_3_O_5_ (M^+^) 381.1325, found 381.1321.

*(Z)-3-(4-(4-Ethoxy-3-methoxybenzylidene)-3-methyl-5-oxo-4,5-dihydro-1H-pyrazol-1-yl)benzoic acid* (**1a**). Solid. m.p. 276–278 ºC. ^1^H-NMR (DMSO): *δ *1.38 (t, 3H), 2.34 (s, 3H), 3.88 (s, 3H), 4.17 (q, 2H), 7.16 (d, 1H), 7.56 (t, 1H), 7.77 (s, 2H), 8.12 (d, 1H), 8.21 (d, 1H), 8.51 (s, 1H), 8.75 (s, 1H), 13.05 (s, 1H); EI-MS m/z 380 (M^+^, 100%); HRMS (EI) m/z calcd. C_21_H_20_N_2_O_5 _(M^+^) 380.1372, found 380.1371.

*(Z)-4-(4-Ethoxy-3-methoxybenzylidene)-3-methyl-1-(4-nitrophenyl)-1H-pyrazol-5(4H)-one *(**1c**)*. *Solid. m.p. 213–215 ºC. ^1^H-NMR (CDCl_3_): *δ* 1.55 (t, 3H), 2.38 (s, 3H), 4.06 (s, 3H), 4.25 (q, 2H), 6.97 (d, 1H), 7.37 (s, 1H), 7.73 (d, 1H), 8.29 (s, 4H), 8.90 (s, 1H); EI-MS m/z 381 (M^+^, 100%); HRMS (EI) m/z calcd. C_20_H_19_N_3_O_5_ (M^+^) 381.1325, found 381.1323.

*(Z)-4-(4-Ethoxy-3-methoxybenzylidene)-3-methyl-1-(4-(trifluoromethyl)phenyl)-1H-pyrazol-5(4H)-one* (**1d**).Solid. m.p. 144–147 ºC. ^1^H-NMR (CDCl_3_): *δ* 1.54 (t, 3H), 2.37 (s, 3H), 4.05 (s, 3H), 4.25 (q, 2H), 6.96 (d, 1H), 7.35 (s, 1H), 7.66 (d, 2H), 7.72 (d, 1H), 8.18 (d, 2H), 8.93 (s, 1H); EI-MS m/z 404 (M^+^, 100%); HRMS (EI) m/z calcd. C_21_H_19_F_3_N_2_O_3_ (M^+^) 404.1348, found 404.1349. 

*(Z)-4-(4-Ethoxy-3-methoxybenzylidene)-3-methyl-1-(3-(trifluoromethyl)phenyl)-1H-pyrazol-5(4H)-one *(**1e**). Solid. m.p. 147–151 ºC. ^1^H-NMR (CDCl_3_): *δ* 1.54 (t, 3H), 2.37 (s, 3H), 4.06 (s, 3H), 4.24 (q, 2H), 6.97 (d, 1H), 7.35 (s, 1H), 7.42 (d, 1H), 7.53 (t, 1H), 7.75 (d, 1H), 8.28 (d, 1H), 8.31 (s, 1H), 8.91 (s, 1H); EI-MS m/z 404 (M^+^, 100%); HRMS (EI) m/z calcd. C_21_H_19_F_3_N_2_O_3_ (M^+^) 404.1348, found 404.1349.

*(Z)-1-(3,5-bis(Trifluoromethyl)phenyl)-4-(4-ethoxy-3-methoxybenzylidene)-3-methyl-1H-pyrazol-5(4H)-one* (**1f**).Solid. m.p. 198–200 ºC. ^1^H-NMR (CDCl_3_): *δ* 1.54 (t, 3H), 2.38 (s, 3H), 4.07 (s, 3H), 4.25 (q, 2H), 6.98 (d, 1H), 7.37 (s, 1H), 7.65 (s, 1H), 7.77 (d, 1H), 8.61 (s, 2H), 8.84 (s, 1H); EI-MS m/z 472 (M^+^, 100%); HRMS (EI) m/z calcd. C_22_H_18_F_6_N_2_O_3_ (M^+^) 472.1222, found 472.1223.

*(Z)-4-(4-Ethoxy-3-methoxybenzylidene)-1-(2-fluorophenyl)-3-methyl-1H-pyrazol-5(4H)-one* (**1g**).Solid. m.p. 153–155 ºC. ^1^H-NMR (CDCl_3_): *δ* 1.52 (t, 3H), 2.34 (s, 3H), 4.00 (s, 3H), 4.22 (q, 2H), 6.93 (d, 1H), 7.22 (q, 2H), 7.32 (t, 1H), 7.36 (s, 1H), 7.52 (t, 1H), 7.67 (d, 1H); EI-MS m/z 354 (M^+^, 100%); HRMS (EI) m/z calcd. C_20_H_19_FN_2_O_3_ (M^+^) 354.1380, found 354.1377.

*(Z)-1-(3,4-Dichlorophenyl)-4-(4-ethoxy-3-methoxybenzylidene)-3-methyl-1H-pyrazol-5(4H)-one* (**1h**). Solid. m.p. 164–166 ºC. ^1^H-NMR (CDCl_3_): *δ* 1.53 (t, 3H), 2.34 (s, 3H), 4.05 (s, 3H), 4.23 (q, 2H), 6.95 (d, 1H), 7.32 (s, 1H), 7.44 (s, 1H), 7.71 (d, 1H), 7.94 (d, 1H), 8.21 (s, 1H), 8.90 (s, 1H); EI-MS m/z 404 (M^+^, 100%); HRMS (EI) m/z calcd. C_22_H_18_F_6_N_2_O_3_ (M^+^) 404.0694, found 404.0695.

*(Z)-1-(3,5-Dichlorophenyl)-4-(4-ethoxy-3-methoxybenzylidene)-3-methyl-1H-pyrazol-5(4H)-one *(**1i**).Solid. m.p. 182–185 ºC. ^1^H-NMR (CDCl_3_): *δ* 1.54 (t, 3H), 2.35 (s, 3H), 4.06 (s, 3H), 4.23 (q, 2H), 6.96 (d, 1H), 7.15 (s, 1H), 7.33 (s, 1H), 7.72 (d, 1H), 8.04 (s, 2H), 8.89 (s, 1H); EI-MS m/z 404 (M^+^, 100%); HRMS (EI) m/z calcd. C_22_H_18_F_6_N_2_O_3_ (M^+^) 404.0694, found 404.0697.

*(Z)-3-(4-(4-Hydroxy-3-methoxybenzylidene)-3-methyl-5-oxo-4,5-dihydro-1H-pyrazol-1-yl)benzoic acid* (**1j**).Solid. m.p. 255–257 ºC. ^1^H-NMR (DMSO): *δ* 2.33 (s, 3H), 3.90 (s, 3H), 6.96 (d, 1H), 7.56 (t, 1H), 7.70 (s, 1H), 7.75 (d, 1H), 8.04 (d, 1H), 8.21 (d, 1H), 8.53 (s, 1H), 8.76 (s, 1H); EI-MS m/z 352 (M^+^, 100%); HRMS (EI) m/z calcd. C_19_H_16_N_2_O_5_ (M^+^) 352.1059, found 352.1057.

*(Z)-3-(4-(3,4-Dihydroxybenzylidene)-3-methyl-5-oxo-4,5-dihydro-1H-pyrazol-1-yl) benzoic acid* (**1k**).Solid. m.p. 278–280 ºC. ^1^H-NMR (DMSO): *δ* 2.34 (s, 3H), 6.93 (d, 1H), 7.57 (t, 1H), 7.65 (s, 1H), 7.76 (d, 1H), 7.92 (d, 1H), 8.18 (d, 1H), 8.52 (s, 1H), 8.63 (s, 1H), 10.45(s,1H); EI-MS m/z 338 (M^+^, 100%); HRMS (EI) m/z calcd. C_19_H_16_N_2_O_5_ (M^+^) 338.0903, found 338.0782.

*(Z)-3-Methyl-4-((5-methylthiophen-2-yl)methylene)-1-(3-nitrophenyl)-1H-pyrazol-5(4H)-one *(**1l**). Solid. m.p. 199–201 ºC. ^1^H-NMR (CDCl_3_): *δ* 2.39 (s, 1H), 2.67 (s, 3H), 7.00 (s, 1H), 7.58 (t, 2H), 7.85 (s, 1H), 8.02 (d, 1H), 8.53 (d, 1H), 8.92 (s, 1H); EI-MS m/z 327 (M^+^, 100%); HRMS (EI) m/z calcd. C_16_H_13_N_3_O_3_S (M^+^) 327.0678, found 327.0675.

*(Z)-4-(4-Ethoxy-3-methoxybenzylidene)-3-methyl-1-phenyl-1H-pyrazol-5(4H)-one* (**1m**).Solid. m.p. 134–139 ºC. ^1^H-NMR (CDCl_3_): *δ* 1.54 (t,3H), 2.36 (s, 3H), 4.05 (s, 3H), 4.23 (q, 2H), 6.95 (d, 1H), 7.19 (t, 1H), 7.32 (s, 1H), 7.42 (t, 2H), 7.72 (d, 1H), 7.97 (d, 2H), 8.98 (s, 1H); EI-MS m/z 336 (M^+^, 100%); HRMS (EI) m/z calcd. C_20_H_20_N_2_O_3_ (M^+^) 336.1474, found 336.1475.

*(Z)-4-(4-Bromobenzylidene)-3-methyl-1-phenyl-1H-pyrazol-5(4H)-one* (**1n**). Solid. m.p. 117–120 ºC. ^1^H-NMR (CDCl_3_): *δ* 2.36 (s, 3H), 7.21 (t, 1H), 7.32 (s, 1H), 7.43 (t, 2H), 7.65 (d, 2H), 7.95 (d, 2H), 8.39 (d, 2H); EI-MS m/z 340 (M^+^, 100%); HRMS (EI) m/z calcd. C_17_H_13_BrN_2_O (M^+^) 340.0211, found 340.0212.

*(Z)-4-(4-Hydroxy-3-methoxybenzylidene)-1-(3-nitrophenyl)-3-phenyl-1H-pyrazol-5(4H)-one* (**1o**). Solid. m.p. 199–202 ºC. ^1^H-NMR (CDCl_3_): *δ* 4.14 (s, 3H), 6.38 (s, 1H), 7.03 (d, 1H), 7.53 (d, 1H), 7.56 (m, 4H), 7.69 (d, 1H), 7.71 (d, 1H), 8.07 (d, 1H), 8.54 (d, 1H), 9.06 (s, 1H), 9.15 (s, 1H); EI-MS m/z 415 (M^+^, 100%); HRMS (EI) m/z calcd. C_23_H_17_N_3_O_5_ (M^+^) 415.1168, found 415.1159. 

*(Z)-3-(4-(4-(Benzyloxy)-3-methoxybenzylidene)-3-methyl-5-oxo-4,5-dihydro-1H-pyrazol-1-yl)benzoic acid(***1p**).Solid. m.p. 249–252 ºC. ^1^H-NMR (DMSO): *δ* 2.35 (s, 3H), 3.89 (s, 3H), 5.26 (s,2H), 7.28–7.50 (m, 7H), 7.57 (t, 1H), 7.77 (d, 2H), 8.14 (d, 1H), 8.22 (d, 1H), 8.52 (s, 1H), 8.77 (s, 1H); EI-MS m/z 442 (M^+^, 100%); HRMS (EI) m/z calcd C_26_H_22_N_2_O_5_ (M^+^) 442.1529, found 442.1530.

*(Z)-3-(4-(3-Methoxy-4-(pentyloxy)benzylidene)-3-methyl-5-oxo-4,5-dihydro-1H-pyrazol-1-yl)benzoic acid* (**1q**).Solid. m.p. 224–226 ºC. ^1^H-NMR (DMSO): *δ* 0.91 (t, 3H), 1.32–1.43 (m, 4H), 1.77 (m, 2H), 2.35 (s, 3H), 3.88 (s, 3H), 4.11 (t, 2H), 7.18 (d,1H), 7.57 (t, 1H), 7.77 (d, 2H), 8.13 (d, 1H), 8.22 (d, 1H), 8.52 (s, 1H), 8.77 (s, 1H); EI-MS m/z 422 (M^+^, 100%); HRMS (EI) m/z calcd. C_24_H_26_N_2_O_5_ (M^+^) 422.1842, found 422.1840.

*(Z)-3-(4-(4-Isopropoxy-3-methoxybenzylidene)-3-methyl-5-oxo-4,5-dihydro-1H-pyrazol-1-yl)benzoic acid* (**1r**).Solid. m.p. 206–208 ºC. ^1^H-NMR (DMSO): *δ* 1.33 (s, 6H), 2.36 (s, 3H), 3.87 (s, 3H), 4.83 (m, 1H), 7,21 (d, 1H), 7.57 (t,1H), 7.76 (d, 1H), 7.79 (s, 1H), 8.15 (d, 1H), 8.22 (d, 1H), 8.53 (s, 1H), 8.75 (s, 1H); EI-MS m/z 394 (M^+^, 100%); HRMS (EI) m/z calcd. C_24_H_26_N_2_O_5_ (M^+^) 394.1529, found 394.1528.

## 4. Conclusions

We have developed a simple, rapid, and efficient one-pot protocol for the preparation of the 4-arylidenepyrazolone derivatives by a solvent-free, microwave-assisted reaction. Furthermore, the procedure used commercially available reagents, giving the desired compounds in good to excellent yields (51–98%). The versatility of this methodology makes it suitable for library synthesis in drug discovery efforts.
